# Prediction of potential small molecule−miRNA associations based on heterogeneous network representation learning

**DOI:** 10.3389/fgene.2022.1079053

**Published:** 2022-12-02

**Authors:** Jianwei Li, Hongxin Lin, Yinfei Wang, Zhiguang Li, Baoqin Wu

**Affiliations:** ^1^ School of Artificial Intelligence, Institute of Computational Medicine, Hebei University of Technology, Tianjin, China; ^2^ Hebei Province Key Laboratory of Big Data Calculation, Hebei University of Technology, Tianjin, China

**Keywords:** small molecule-miRNA association prediction, heterogeneous information, heterogeneous network representation learning, machine learning, lightgbm

## Abstract

MicroRNAs (miRNAs) are closely associated with the occurrences and developments of many complex human diseases. Increasing studies have shown that miRNAs emerge as new therapeutic targets of small molecule (SM) drugs. Since traditional experiment methods are expensive and time consuming, it is particularly crucial to find efficient computational approaches to predict potential small molecule-miRNA (SM-miRNA) associations. Considering that integrating multi-source heterogeneous information related with SM-miRNA association prediction would provide a comprehensive insight into the features of both SMs and miRNAs, we proposed a novel model of Small Molecule-MiRNA Association prediction based on Heterogeneous Network Representation Learning (SMMA-HNRL) for more precisely predicting the potential SM-miRNA associations. In SMMA-HNRL, a novel heterogeneous information network was constructed with SM nodes, miRNA nodes and disease nodes. To access and utilize of the topological information of the heterogeneous information network, feature vectors of SM and miRNA nodes were obtained by two different heterogeneous network representation learning algorithms (HeGAN and HIN2Vec) respectively and merged with connect operation. Finally, LightGBM was chosen as the classifier of SMMA-HNRL for predicting potential SM-miRNA associations. The 10-fold cross validations were conducted to evaluate the prediction performance of SMMA-HNRL, it achieved an area under of ROC curve of 0.9875, which was superior to other three state-of-the-art models. With two independent validation datasets, the test experiment results revealed the robustness of our model. Moreover, three case studies were performed. As a result, 35, 37, and 22 miRNAs among the top 50 predicting miRNAs associated with 5-FU, cisplatin, and imatinib were validated by experimental literature works respectively, which confirmed the effectiveness of SMMA-HNRL. The source code and experimental data of SMMA-HNRL are available at https://github.com/SMMA-HNRL/SMMA-HNRL.

## Introduction

MicroRNAs (miRNAs) are a large group of non-coding RNAs (ncRNAs) with approximately 22 nucleotides in length, which are widespread in eukaryotes (Bartel, 2004). Since the discovery of the first miRNA, lin-4, in Caenorhabditis elegans by Lee et al. (1993), accumulating evidence has demonstrated that miRNAs play vital roles in various key physiological processes, including cell proliferation (Cheng et al., 2005), cell differentiation (Miska, 2005), cell apoptosis (Xu et al., 2004), regulation of animal immune function (Stern-Ginossar et al., 2007) and regulation of gene expression levels (Shivdasani, 2006) etc. Meanwhile, many studies have confirmed that numerous complex human diseases are also closely related to the dysregulations of related key miRNAs (Croce and Calin, 2005; Sayed and Abdellatif, 2011; Chen et al., 2019). Due to the ubiquity of miRNAs in physiological and pathological processes, miRNAs are also recognized as a potentially important class of drug targets (Liu et al., 2008; Rossi, 2009; Cheng et al., 2015). Nowadays, computer-aided drug design has been applied broadly in the early stages of drug development, and the prediction results of computational models can provide directions for researchers to find the most effective drugs, reduce experimental costs and blindness significantly (Zhao et al., 2019). Among them, computational prediction of Small Molecule-miRNA (SM-miRNA) associations is a critical step in drug R&D (Chen et al., 2018). With deepening research in the field of SM-miRNA association prediction, many corresponding databases have been constructed, such as SM2miR (Liu et al., 2013), NoncoRNA (Li et al., 2020), mTD (Chen et al., 2017a), and NRDTD (Chen et al., 2017b). These databases provide abundant resources for exploring SM-miRNA associations and make it possible to construct effective and accurate SM-miRNA association prediction models. SM-miRNA computational models are usually divided into three categories, models based on biological networks, models based on machine learning algorithms and other prediction models.

In the first category, the models construct biological networks based on biological information and utilize network topology information to predict potential SM-miRNA associations. In 2015, Lv et al. (2015) built an integrated heterogeneous network by SM and miRNA similarity networks and SM-miRNA association network. They employed random walk with restart (RWR) algorithms on the heterogeneous network for predicting potential SM-miRNA associations. In 2016, Li et al. (2016) developed a network-based inference framework which was termed SMiR-NBI. A heterogeneous network which consisted of SMs, miRNAs and genes was constructed, and the network-based inference algorithm was implemented to calculate the association scores between the given SMs and miRNAs. Qu et al. (2018) proposed a prediction framework based on a heterogeneous network which was named TLHNSMMA in 2018. TLHNSMMA constructed a triple-layer network and finally predicted potential SM-miRNA associations by the iterative update algorithm based on the global network. In the same year, Guan et al. (2018) proposed GISMMA based on Graphlet interactions. Graphlet interactions between SMs and miRNAs were calculated on SM and miRNA similarity networks. In 2020, Shen et al. (2020) proposed a computational model named SMMART based on graph regularization technique.

The second category of the computational models predicts novel SM-miRNA associations based on machine learning algorithms. Extracting the biological features of SMs and miRNAs for training the classifiers, potential associations are predicted with machine learning algorithms. In 2019, Wang et al. (2019) proposed RFSMMA model based on random forest algorithm. A filtering approach was employed to extract reliable features of SM-miRNA pairs by using their similarity data. Subsequently, the features were exploited to train the random forest model, and potential SM-miRNA associations were predicted with it. In 2022, Wang et al. (2022) developed an EKRRSMMA model based on ensemble of kernel ridge regression. By constructing different feature subsets for SMs and miRNAs, an integrated learning model containing multiple KRR-based base learning tasks was constructed. The prediction results of all base learners were averaged and the results were introduced as the SM-miRNA association scores. Beside the above two categories, there are also other models which can predict SM-miRNA associations. In 2019, Xie et al. (2019) proposed a new text mining framework, termed EmDL, for extracting associations between miRNAs and SMs efficacy from the literature and recording them in the database. In 2012, Jiang et al. (2012) constructed a SM-miRNA Network (SMirN) for each type of 23 common cancers. The associations of cancer-related miRNAs with SMs were determined by the enrichment scores. To give readers a clear overview, Supplementary Material S1 summarizes the aforementioned models in a tabular form.

More recently, network representation learning algorithms have been widely used in the field of biomedical sciences (Yue et al., 2020). In 2021, Thafar et al. (2021) predicted drug-target associations with node2vec (Grover and Leskovec, 2016) and ensemble learning. Ji et al. (2020) adopted the LINE (Tang et al., 2015) to catch the feature information from the drug-target network and utilized random forest method as the classifier in 2020. Early network representation learning algorithms could only address homogeneous networks. Yet in the reality, a vast number of networks are composed of different types of entities and different kinds of relationships, which were called heterogeneous information networks. Heterogeneous network representation learning algorithm was more capable of retaining the rich structural and semantic information in heterogeneous information networks. Thus, numerous heterogeneous network representation learning algorithms have been proposed rapidly, and have been implemented into biological networks. In 2021, Deng et al. (2021) used HIN2Vec (Fu et al., 2017) to learn the embedding vectors for each node in lncRNA-disease-miRNA heterogeneous network and utilized gradient boosting tree (GBT) classifier for predicting potential lncRNA-disease associations. In 2018, Zhu et al. (2018) utilized Metapath2vec (Dong et al., 2017) to extract heterogeneous network features and employ a kernelized Bayesian matrix factorization method for predicting drug-gene associations.

In this study, we proposed a novel model, SM-MiRNA Association prediction based on Heterogeneous Network Representation Learning (SMMA-HNRL), to improve the performance of SM-miRNA association prediction. The data was collected from six networks (miRNA-SM, miRNA-disease, miRNA-miRNA, SM-disease, SM-SM, disease-disease) for constructing miRNA-SM-disease heterogeneous information network. Inspired by the success of integrated features on the lncRNA-disease association prediction problem (Li et al., 2021), we employed two excellent heterogeneous network representation learning algorithms, HIN2Vec (Fu et al., 2017) and HeGAN (Hu et al., 2019), to embed all nodes of the miRNA-SM-disease heterogeneous network into low-dimensional vectors respectively, and then combined them into the novel feature vectors of SMs and miRNAs. Finally, Hadamard function was chosen to gain all SM-miRNA vector pairs, and LightGBM (Ke et al., 2017) classifier was selected to predict potential SM-miRNA associations. To assess the prediction performance of SMMA-HNRL, we compared it with three state-of-the-art models with 10-fold-cross validations. For validating the robustness, our model performed on two independent validation datasets. Moreover, the dependable prediction performance of SMMA-HNRL was also confirmed with three case studies. All the results of evaluation experiments demonstrated the reliable and predictive performance of SMMA-HNRL.

## Materials and methods

### SM-miRNA association network

The experimentally validated SM-miRNA associations used in our study was downloaded from the SM2miR v3.0 database (Liu et al., 2013). By manual inspection, we eliminated the SMs which were not present in DrugBank (Wishart et al., 2018), and merged the mature miRNAs which were generated from the same precursor miRNAs (e.g., hsa-miR-21-3p and hsa-miR-21-5p). Then, the format of mature miRNAs was converted to that of precursor miRNA. Moreover, non-human data and duplicate SM-miRNA associations were culled out. Finally, 1766 experimentally validated SM-miRNA associations which included 546 miRNAs and 93 SMs were obtained. Finally, an SM-miRNA association network was constructed based on these 1766 associations which was used during training the model and the cross-validation evaluation.

### miRNA-disease association network

Human experimentally validated miRNA-disease associations was downloaded from the HMDD v3.2 database (Huang et al., 2019), and disease names of miRNA-disease associations were converted into the standardized names according to the MESH glossary. After removing duplicated data, a total of 18,732 miRNA-disease associations involving 1206 miRNAs and 892 diseases were obtained and the miRNA-disease association network was constructed with them.

### SM-disease association network

The SM-disease association data was collected from the SCMFDD-L dataset in the SCMFDD database (Zhang et al., 2018). SCMFDD acquired available drug-disease associations from the CTD database (Davis et al., 2017) and selected drugs with known drug substructure information. The SM drugs were selected and duplicated data was removed. Through screening, 49,032 pairs of SM-disease associations were obtained which included 1313 SMs and 2822 diseases.

### Integrated SM similarity network

The integrated SM similarity data was downloaded from the DrugSimDB database (Azad et al., 2021) which were the mean values of chemical structure similarity, target protein sequence-based similarity, target protein functional similarity and drug-induced pathway similarity of SM drugs. The integrated SM similarity network was constructed according to this data which included 1331 SM drugs.

### Integrated miRNA similarity network

The miRNA similarity data was sourced from miRNA-disease associations and Gene Ontology (GO) annotations of miRNA target genes respectively. In 2010, Wang et al. (2010) proposed a method named MISIM to calculate the functional similarity of miRNAs based on the hypothesis that functionally similar miRNAs were often associated with semantically similar diseases. In 2019, MISIM was updated and named MISIM v2.0 by our research group which not only had a threefold increase in data content compared with MISIM but also improved the original MISIM algorithm (Li et al., 2019). Additionally, Yang et al. (2018) developed a novel method called MIRGOFS which calculated the functional similarity of miRNAs based on the GO annotations of their target genes. We downloaded miRNA similarity data from MISIM v2.0 and the normalized miRNA similarity network data from MIRGOFS respectively. To facilitate calculation, mature miRNAs produced from the same pre-miRNA were merged and converted into the precursor miRNA.

Inspired by SM similarity network, we integrated the above two miRNA similarity networks with average ensemble method. If one miRNA was in only one similarity network, its similarity value was considered the final results. In the end, an integrated miRNA similarity network consisting of 1309 miRNAs was obtained.

### Disease semantic similarity network

The semantic similarity values between two diseases can be calculated based on the Medical Subject Headings (MESH) disease structure. Each disease represented by the MESH descriptor, which were obtained from National Library of Medicine (https://www.nlm.nih.gov/), could be represented as a Directed Acyclic Graph (DAG). One disease d can be denoted in the DAG as follows:

$$DAG_{d}=\left(d,T_{d},E_{d}\right)$$ (1)

Where $T_{d}$ represented the node set which was composed of disease d and all its ancestor nodes; and $E_{d}$ represented the edge set of disease d in the DAG. The semantic contribution value D of disease t to disease d can be defined by the following equation:

$$\left\{\begin{array}{c} D_{d}\left(t\right)=1\\ D_{d}\left(t\right)=\max\left\{\Delta^{*}D_{d}\left(t^{\prime }\right)|t^{\prime }\in children\;of\;t\right\}\;if\;t\neq d \end{array}\right.$$ (2)

Eq. (2) indicated that if there were multiple paths for disease t to reach disease d in the DAG graph, the shortest path needed to be selected to achieve the maximum semantic contribution value. ∆ was the semantic contribution factor which reflected the influence degree of the parent node on the child nodes in the DAG graph. Based on the related study by Xuan et al. (2013), the value of ∆ was set as 0.5 in the beginning of the calculation. After accumulating the semantic contribution values of all disease nodes in the DAG, the semantic contribution value of every disease was obtained.

$$DV\left(d\right)=\underset{t\in T_{d}}{\sum }\;D_{d}\left(t\right)$$ (3)

With the semantic contribution value of each disease, we could calculate the similarity between any two diseases $d_{i}$ and $d_{j}$ according to Eq. (4).

$$Sim\left(d_{i},d_{j}\right)=\frac{\underset{t\in T_{d_{i}}\cap T_{d_{j}}}{\sum }\;\left(D_{d_{i}}\left(t\right)+D_{d_{j}}\left(t\right)\right)}{DV\left(d_{i}\right)+DV\left(d_{j}\right)}$$ (4)

where t was used to denote nodes of disease $d_{i}$ and $d_{j}$ in the DAG structure, $DV\left(d_{i}\right)$ and $DV\left(d_{j}\right)$ represented semantic values of disease $d_{i}$ and $d_{j}$ , and $D_{d_{i}}\left(t\right)$ and $D_{d_{j}}\left(t\right)$ were indicated as the semantic contributions of disease t to diseases $d_{i}$ and $d_{j}$ .

### Feature extraction by HeGAN

HeGAN was the first method which introduced Generative Adversarial Networks (GAN) into heterogeneous networks representation learning problem (Hu et al., 2019). The basic idea of GAN was to train the discriminator and the generator with the ideas of competition, and thus obtained the data latent distribution. Compared with traditional heterogeneous network representation learning methods, HeGAN exhibited more stability to sparse data or noisy data and achieved the best performance in downstream tasks on public datasets. Besides, it should be noted that HeGAN did not employ meta-paths and there was no costly meta-path setup.

**FIGURE 1:**
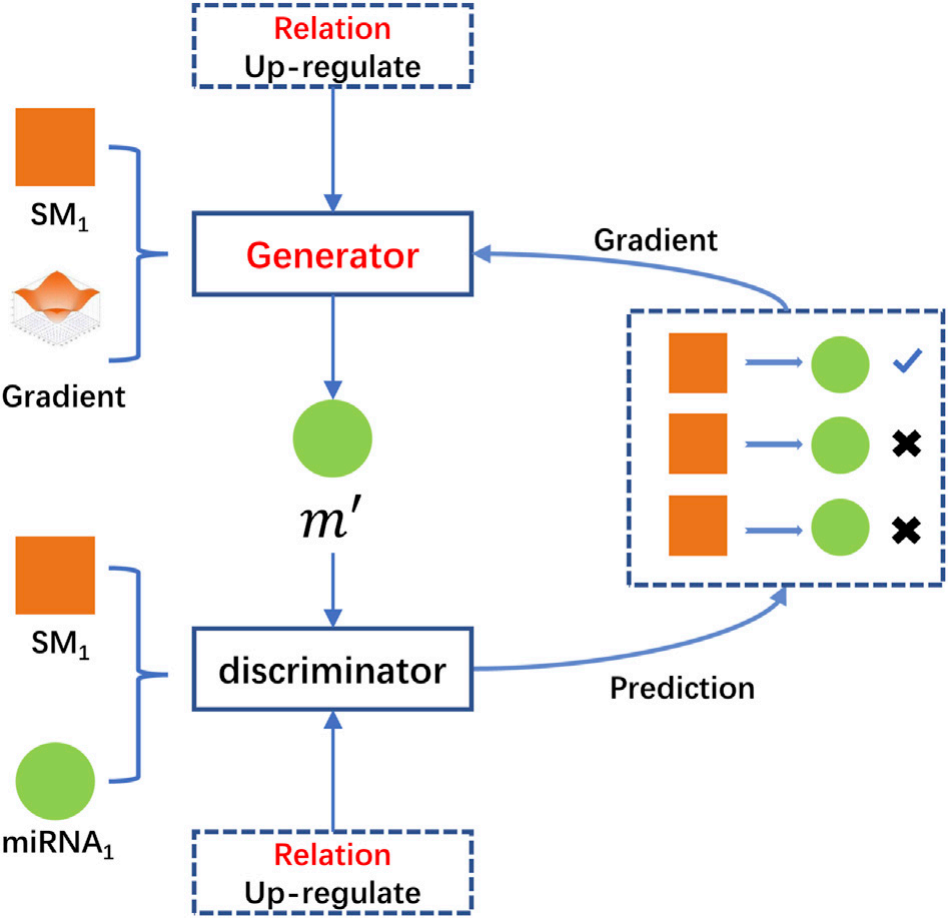
The framework of the HeGAN algorithm.

HeGAN was composed of two main competing modules, the relational perception discriminator and the generalized generator. For a given node, the generalized generator firstly attempted to generate fake samples associated with the given node and fed these fake samples to the discriminator. The discriminator accepted true samples from the real network and fake samples generated by the generator, respectively. Secondly, HeGAN attempted to adjust its parameters to separate the fake samples from the true samples repeatedly. Finally, the discriminator predicted the probability of two nodes that there was a relationship r between them. In the iterative process, the trained discriminator continually forced the generator to generate better fake samples, while the discriminator would enhance its judgment ability correspondingly. Figure 1. illustrates the framework of the HeGAN algorithm.

Traditional network representation learning methods were limited performance due to lack of making full use of the valuable semantic information of heterogeneous information networks. For a given miRNA *m*, suppose that there were two nodes *SM*_*1*_ and *SM*_*2*_ which were associated with it. The traditional methods simply regarded *SM*_*1*_ and *SM*_*2*_ as true nodes and did not analyze them in depth. Normally, *SM*_*1*_ and *SM*_*2*_ were generally associated with *m* due to multiple reasons, such as *SM*_*1*_ upregulated *m*, while *SM*_*2*_ downregulated *m.* The traditional methods did not take full use of the valuable semantics embedded in heterogeneous networks, which would lower the accuracy of functional predictions of *SM*_*1*_ and *SM*_*2*_. The relational perception discriminator and generalized generator introduced by HeGAN were more suitable for distinguishing various types of semantic relations between two nodes. Besides that, the negative samples of the traditional methods were limited to the number of known samples. In practice, the most representative negative samples were likely to be located between the embedding vectors corresponding to existing nodes, not the existing nodes. To better generate negative samples, HeGAN specifically introduced one generalized generator to generate negative nodes which did not exist in the samples. For example, $m^{\prime }$ in Figure 1, it did not exist in the original graph, but it was the node which best represented the original network.

### Feature extraction by HIN2Vec

**FIGURE 2:**
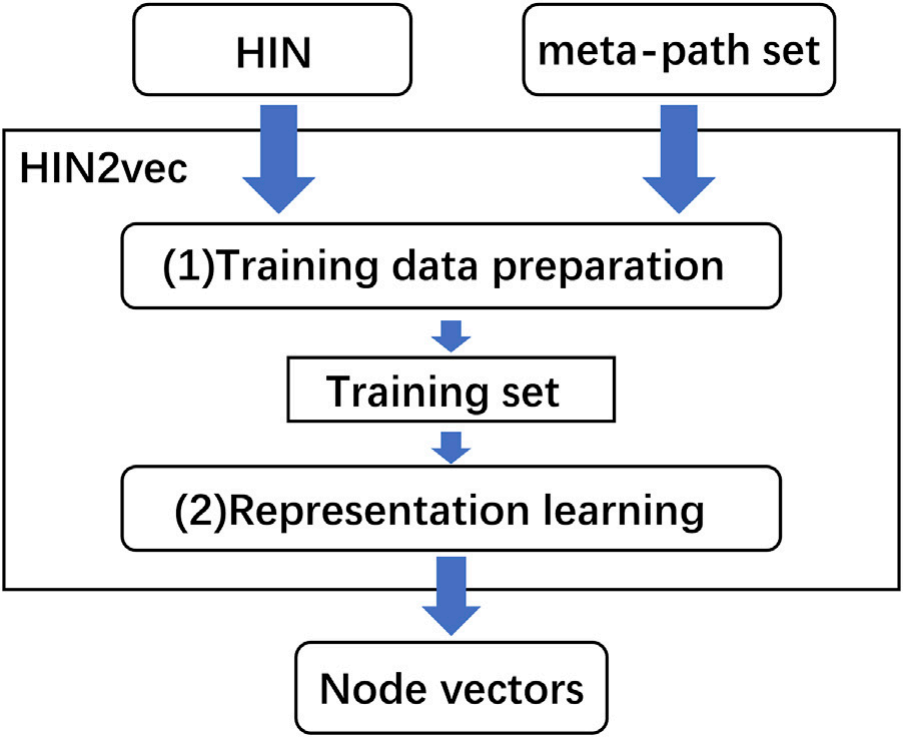
The flowchart of HIN2Vec algorithm.

HIN2Vec was another heterogeneous network representation learning method with excellent performance (Fu et al., 2017). The core part of HIN2Vec was one three-layer neural network which learned the rich information from the heterogeneous information network by captured different relationship information of network topologies. HIN2Vec not only obtained low-dimensional representations of nodes, but also learned representations of relationships (meta-paths) in the networks. HIN2Vec also got the best performance in downstream tasks. The flowchart of the HIN2Vec algorithm is shown in Figure 2.

As illustrated in Figure 2, the HIN2Vec model consisted of two main parts, one was the training data preparation which was generated based on random walk and negative sampling, the other was representation learning which was performed on the generated training data. In training data generation, HIN2Vec represented the heterogeneous network in the form of $\left\langle m,n,r,L\left(m,n,r\right)\right\rangle$ , where m,n represented two nodes, r was a different type of relationship between two nodes, $L\left(m,n,r\right)$ was a binary value representing whether there was a relationship between the m and n nodes. HIN2Vec utilized random walk algorithm to generate node sequences and differentiated their types r . It was unlike Metapath2vec (Dong et al., 2017) which walked exactly according to a given meta-path, the HIN2Vec model completely randomly selected different walking nodes. If there was a connection between two nodes, a random walk could be conducted. Considering the above, HIN2Vec would retain more contextual information and acquire richer semantics.

**FIGURE 3:**
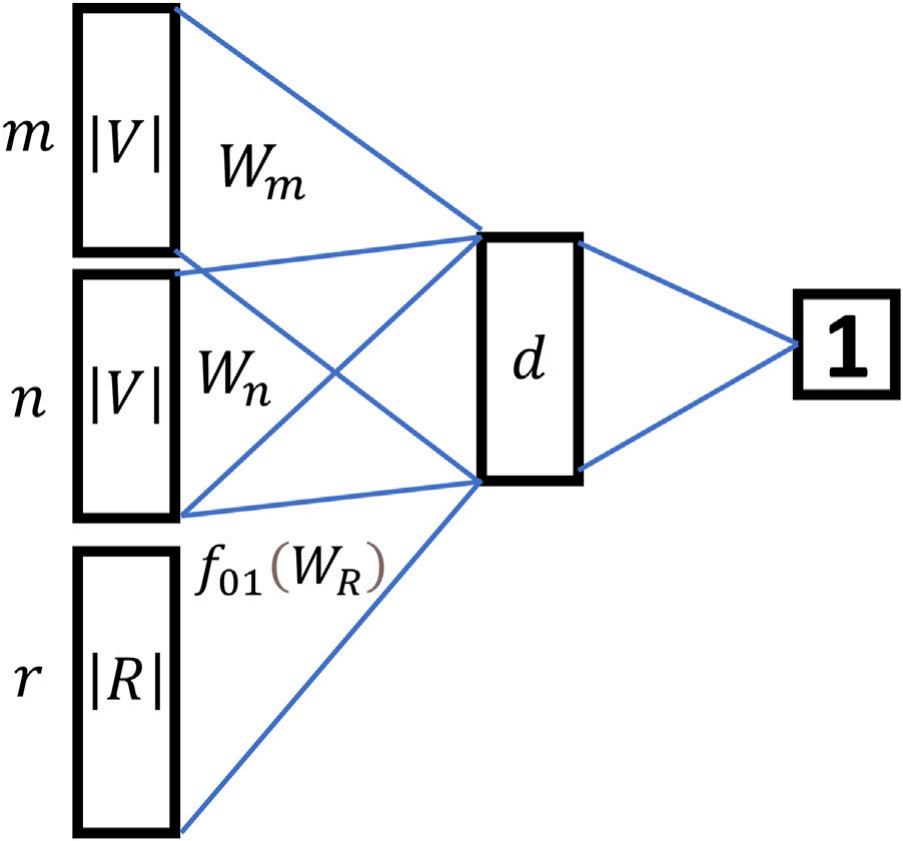
The neural network structure of HIN2Vec.

In the representation learning part, HIN2Vec innovatively transformed the relationship between two nodes from multi-classification problem to a multiple binary classification problem. HIN2Vec built a three-layer feedforward neural network as a logical binary classifier to predict whether there is a definite relationship r between two nodes which avoided traversing all relationships in the network, and learned the vector representation of nodes and relationships at the same time. Figure 3 showed the neural network structure of HIN2Vec.

From Figure 3, the relationship between two nodes in HIN2Vec was no longer considered as a prediction object, but as training data of the input layer. The model mainly predicted whether there was a specific relationship r between node m and node n .The inputs to the model were three one-hot vectors, $\vec{m},\vec{n}$ and $\vec{r}$ . They were converted in the latent layer to the latent vector $W_{M}^{\prime }\vec{m},W_{N}^{\prime }\vec{n}$ and $f_{01}\left(W_{R}^{\prime }\vec{r}\right)$ . Since the semantic information of the node was different from the semantic information of the relationship, the regularization function $f_{01}\left(.\right)$ was added before the relationship r for regularization to ensure that the value of the relationship r was between 0 and 1. Then the three latent vectors were aggregated with the Hadamard function (the elements in the vector were multiplied two by two) to obtain the form of $W_{M}^{\prime }\vec{m}⊙W_{N}^{\prime }\vec{n}⊙f_{01}\left(W_{R}^{\prime }\vec{r}\right)$ , and applied the identity function to activate. At the output layer, HIN2Vec took summation for the output d-dimensional vectors in the hidden layer and activated them with the Sigmoid function. Eventually, $sigmoid\left(\sum W_{M}^{\prime }\vec{m}⊙W_{N}^{\prime }\vec{n}⊙f_{01}\left(W_{R}^{\prime }\vec{r}\right)\right)$ was utilized for logical classification.

HIN2Vec was trained iteratively on the training set D with a backpropagation algorithm with stochastic gradient descent. By continuously adjusting the weights of each entry $W_{m}$ , $W_{n}$ and $W_{R}$ in set D, the objective function O was maximized, which was the multiplication of each training data entity $O_{m,n,r}\left(m,n,r\right)$ in set D. To simplify computation, HIN2Vec maximizes log⁡⁡O instead of directly maximizing O . The objective functions O and log⁡⁡O were defined as follows:

$$\begin{array}{c} O\propto \log O=\underset{m,n,r\in D}{\sum }\;\log O_{m,n,r}\left(m,n,r\right) \end{array}$$ (5)

In particular, in a training sample $\left\langle m,n,r,L\left(m,n,r\right)\right\rangle$ , if $L\left(m,n,r\right)$ was 1, $O_{m,n,r}\left(m,n,r\right)$ aimed to maximize $P\left(r|m,n\right)$ . Otherwise, the $O_{m,n,r}\left(m,n,r\right)$ aimed to minimize $P\left(r|m,n\right)$ . $O_{m,n,r}\left(m,n,r\right)$ , $\log O_{m,n,r}\left(m,n,r\right)$ and $P\left(r|m,n\right)$ were derived by the following formula:

$$\begin{array}{c} O_{m,n,r}\left(m,n,r\right)=\left\{\begin{array}{c} P\left(r|m,n\right),\;if\;L\left(m,n,r\right)=1\\ 1-P\left(r|m,n\right),\;if\;L\left(m,n,r\right)=0 \end{array}\right. \end{array}$$ (6)

$$\begin{array}{c} \log O_{m,n,r}\left(m,n,r\right)=L\left(m,n,r\right)\log P\left(r|m,n\right)\\ +\left[1-L\left(m,n,r\right)\right]\log\left[1-P\left(r|m,n\right)\right] \end{array}$$ (7)

$$\begin{array}{c} P\left(r|m,n\right)=sigmoid\left(\sum W_{m}^{\prime }\vec{m}⊙W_{n}^{\prime }\vec{n}⊙f_{01}\left(W_{R}^{\prime }\vec{r}\right)\right) \end{array}$$ (8)

Then, HIN2Vec adjusted the weights of $W_{M}^{\prime }\vec{m},W_{N}^{\prime }\vec{n}$ and $W_{R}^{\prime }\vec{r}$ according to the gradients of $\log O_{m,n,r}\left(m,n,r\right)$ differentiated by $W_{M}^{\prime }\vec{m},W_{N}^{\prime }\vec{n}$ and $W_{R}^{\prime }\vec{r}$ , and thus maximized the objective function O . The specific definitions were as follows:

$$\begin{array}{c} W_{M}^{\prime }\vec{m}≔W_{M}^{\prime }\vec{m}+\frac{dlogO_{m,n,r}\left(m,n,r\right)}{dW_{M}^{\prime }\vec{m}} \end{array}$$ (9)

$$\begin{array}{c} W_{N}^{\prime }\vec{n}≔W_{N}^{\prime }\vec{n}+\frac{dlogO_{m,n,r}\left(m,n,r\right)}{dW_{N}^{\prime }\vec{n}} \end{array}$$ (10)

$$\begin{array}{c} W_{R}^{\prime }\vec{r}≔W_{R}^{\prime }\vec{r}+\frac{dlogO_{m,n,r}\left(m,n,r\right)}{dW_{R}^{\prime }\vec{r}} \end{array}$$ (11)

### Feature vector merging

With heterogeneous network representation learning method HeGAN and HIN2Vec based on the above generative adversarial network and meta-path random walk, we obtained two feature vector matrices, U and V , respectively. The final merging feature matrix X used in SMMA-HNRL was expressed with the following merging formula:

$$\begin{array}{c} X=\left[U,V\right] \end{array}$$ (12)

where   represented the vector connect operation.

### SMMA-HNRL model

**FIGURE 4:**
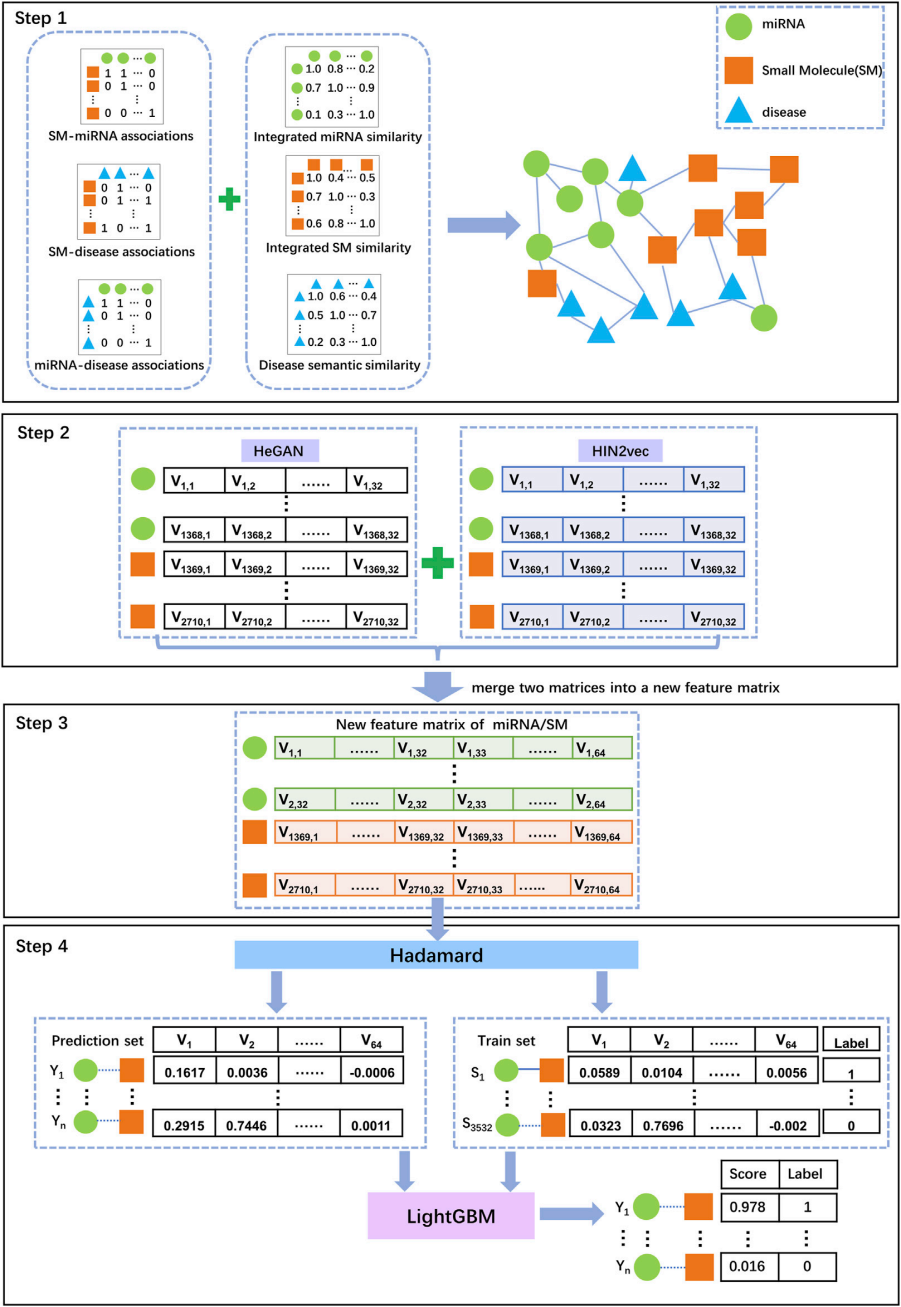
The flowchart of SMMA-HNRL model. Step 1: The associated data and similarity data obtained from different biological databases were preprocessed, a heterogeneous information network was constructed with three association networks (miRNA-SM, miRNA-disease, SM-disease) and three similarity networks (miRNA-miRNA, SM-SM, disease-disease) in our study. Step 2: With two different network representation learning algorithms, HeGAN and HIN2Vec, two feature matrices of the heterogeneous information networks were obtained. Step 3: Combining the feature vectors of miRNAs and SMs from two feature matrices, a merged feature matrix was finally obtained. Step 4: Hadamard function was adopted to convert SM and miRNA feature vectors into a feature vector for a SM-miRNA pair. The known SM-miRNA associations from the heterogeneous information network were chosen as training set for LightGBM classifier to predict potential SM-miRNA associations.

In this study, we developed a novel model termed SMMA-HNRL to improve the performance of predicting potential SM-miRNA associations. The flowchart of SMMA-HNRL was shown in Figure 4.

## Results

### Evaluation metrics

The Recall, Precision, Accuracy, F1 Score, ROC curve with AUC (area under ROC curve) (Obuchowski and Bullen, 2018) value and PR curve with AUPR (area under PR curve) (Saito and Rehmsmeier, 2015) value were adopted as indicators for evaluating the performance of SMMA-HNRL. In contrast experiments, the average AUC values and the AUPR values of ten training sets of each model were calculated and the corresponding ROC curves and PR curves were drawn according to the results of 10-fold cross validation. Finally, the average values of all evaluation indicators were used to evaluate the model performance. For experiment details, please see Supplementary Material S2.

### Comparison with other feature vector merging methods

After we obtained two sets of feature vectors of SM and miRNA by HeGAN and HIN2Vec, we conducted comparative experiments to evaluate the performances of different merging methods. Three merging methods (connection, averaging, and multiplication) were adopted to fuse two feature vectors of both SM and miRNA into one integrated vector. According to the experimental results, the connect operation had obtained the best performance. The detailed experimental results are shown in Table 1.

**TABLE 1:** The six evaluation metrics results of the three merging methods.

	Recall	Precision	Accuracy	F1 score	AUC	AUPR
Connection	0.9745	0.9563	0.9649	0.9652	0.9875	0.9885
Averaging	0.9513	0.9456	0.9482	0.9483	0.9828	0.9813
Multiplication	0.9439	0.9491	0.9465	0.9463	0.9814	0.9823

### Classifier selection

**FIGURE 5:**
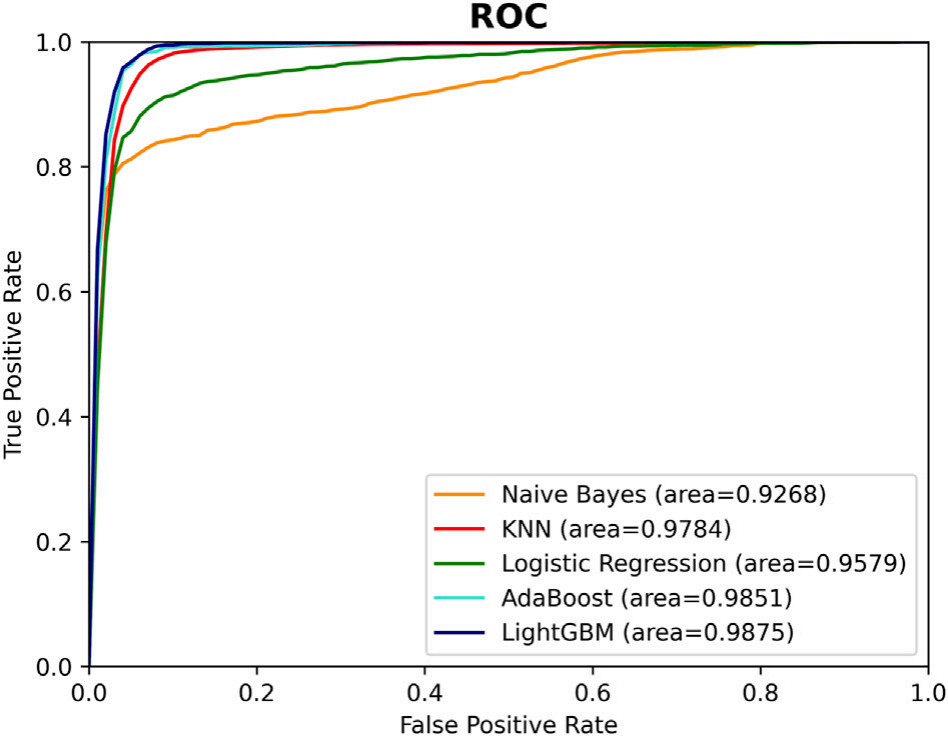
The ROC curves of different classifiers.

After calculating of the SM-miRNA pair vectors, the problem of predicting potential SM-miRNA associations could be considered as a binary classification problem. In our study, 1766 pairs of SM-miRNA associations were downloaded from the SM2miR database as positive samples, and the same amount of SM-miRNA associations from all remaining combinations were randomly selected as negative samples. During the classifier selection, five different popular machine learning methods, Naive Bayes (NB) (Yang, 2018), Linear Regression (LR) (Maulud and Abdulazeez, 2020), K-Nearest Neighbor (KNN) (Zhang and Zhou, 2007), AdaBoost (Freund and Schapire, 1997) and LightGBM (Ke et al., 2017), were tested based on the merging feature vectors of the above samples, respectively. The performance of these five classifiers was evaluated with the Recall, Precision, Accuracy, F1 Score, AUC, and AUPR. Figure 5; Supplementary Material S3 illustrated the performance of these classifiers.

### Vector function selection for SM-miRNA pair

After gaining node vectors of SMs and miRNAs by the heterogeneous network representation learning algorithms, SM-miRNA pair vectors were subsequently calculated with vector functions. We study four commonly functions, Hadamard, Average, Minus and Absolute Minus (Deng et al., 2021), which merged one SM vector and one miRNA vector into one SM-miRNA pair vector. 10-fold cross validations of SMMA-HNRL with these four functions were employed in turn. Table 2 documented the descriptions of SM-miRNA pair vector functions and the corresponding AUC values of 10-fold cross validations. The experimental results demonstrated that Hadamard function outperformed the remaining three vector combinations. It could better remain the association between one SM vector and one miRNA vector. Therefore, Hadamard function was chosen as the SM-miRNA pair vector function for the following experiments.

**TABLE 2:** Vector functions and AUC values of 10-fold cross validations.

Functions	Hadamard	Average	Minus	Absolute minus
Descriptions	$\vec{v_{1i}}*\vec{v_{2i}}$	$\frac{\vec{v_{1i}}+\vec{v_{2i}}}{2}$	$\vec{v_{1i}}-\vec{v_{2i}}$	$\left|\vec{v_{1i}}-\vec{v_{2i}}\right|$
AUC	0.9875	0.9829	0.9808	0.9701

### Parameter tuning

In SMMA-HNRL, HeGAN, and HIN2Vec were utilized for feature extraction respectively, which are both highly encapsulated representation learning models. In our study, most of the inner parameters of HeGAN and HIN2Vec were set to the recommended values, and the number of dimensions of features was treated as hyperparameters. The 16-dimensional, 32-dimensional, 64-dimensional and 128-dimensional topological feature vectors of SM nodes and miRNA nodes were calculated and merged respectively for gaining the optimal combination of feature vector dimensions. The results of 10-fold cross validation of different combinations were shown in Table 3. The numbers in the vector combination name represented feature vector dimensions, for example, HeGAN16HIN2V16 represented the integrated features combined with 16 dimensional features of HeGAN and 16 dimensional features of HIN2Vec. In Table 3, the best evaluation results of each metric were in bold. With a comprehensive consideration, HeGAN32HIN2V32 achieved the best results in the most evaluation metrics which was also marked in bold. Finally, HeGAN32HIN2V32 was selected as the final feature combination of SMMA-HNRL.

**TABLE 3:** The six evaluation metrics results of different vector combinations.

	Recall	Precision	Accuracy	F1 score	AUC	AUPR
HeGAN16HIN2V16	0.9355	0.9550	0.9456	0.9450	0.9826	0.9839
HeGAN16HIN2V32	0.9575	0.9582	0.9578	0.9578	0.9865	0.9880
HeGAN16HIN2V64	0.9541	0.9575	0.9558	0.9557	0.9853	0.9882
HeGAN16HIN2V128	0.9541	0.9559	0.9550	0.9549	0.9858	0.9866
HeGAN32HIN2V16	0.9626	0.9514	0.9567	0.9569	0.9850	0.9861
**HeGAN32HIN2V32**	0.9745	0.9563	**0.9649**	**0.9652**	**0.9875**	0.9885
HeGAN32HIN2V64	0.9711	0.9529	0.9615	0.9619	0.9871	0.9886
HeGAN32HIN2V128	**0.9774**	0.9522	0.9640	0.9645	0.9868	0.9875
HeGAN64HIN2V16	0.9626	0.9514	0.9567	0.9569	0.9850	0.9861
HeGAN64HIN2V32	0.9632	0.9514	0.9570	0.9572	0.9859	0.9843
HeGAN64HIN2V64	0.9677	0.9586	0.9629	0.9631	0.9867	0.9873
HeGAN64HIN2V128	0.9694	0.9587	0.9638	0.9639	0.9870	0.9862
HeGAN128HIN2V16	0.9496	0.9546	0.9522	0.9520	0.9861	0.9868
HeGAN128HIN2V32	0.9615	**0.9606**	0.9609	0.9610	0.9874	**0.9890**
HeGAN128HIN2V64	0.9660	0.9580	0.9618	0.9619	0.9868	0.9879
HeGAN128HIN2V128	0.9643	0.9580	0.9609	0.9611	0.9869	0.9859

### Ablation study

One of the significant characteristics of SMMA-HNRL is that the node feature vectors which were obtained from HeGAN and HIN2Vec are merged. To explore whether merging node features is effective for predicting SM-miRNA associations, we designed the ablation studies to evaluate the performance of the methods with only HeGAN feature vectors, only HIN2Vec feature vectors, and the merging of HeGAN feature vectors and HIN2Vec feature vectors. They were named as HeGAN32, HIN2V32, and HeGAN32HIN2V32 respectively. The results of different feature vector methods were shown in Table 4. It can be seen from Table 4 that HeGAN32HIN2V32 outperformed the other methods.

**TABLE 4:** Ablation study results of different feature vector models.

	Recall	Precision	Accuracy	F1 score	AUC	AUPR
HeGAN32	0.9485	0.9471	0.9476	0.9476	0.9826	0.9822
HIN2V32	0.9417	0.9503	0.9462	0.9459	0.9825	0.9840
HeGAN32HIN2V32	0.9745	0.9563	0.9649	0.9652	0.9875	0.9885

**FIGURE 6:**
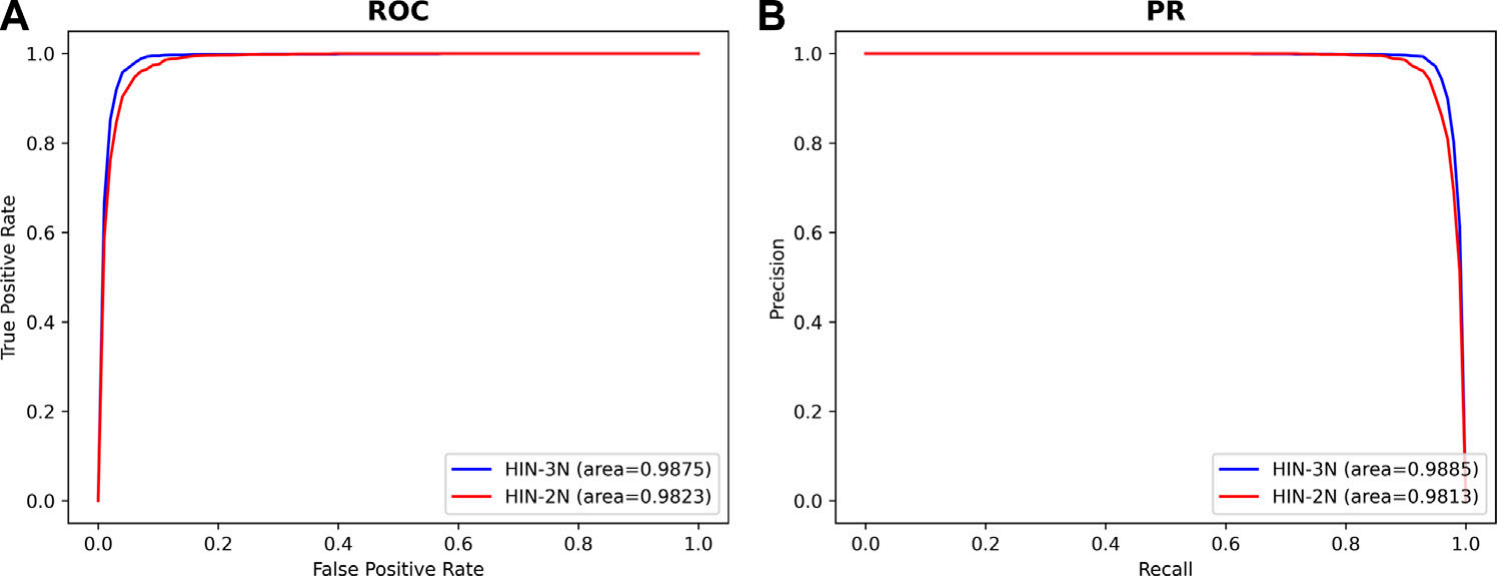
The ROC curves **(A)** and PR curves **(B)** on different heterogeneous networks.

In order to confirm the hypothesis that adding disease association information can increase the information richness between miRNAs and SMs which would improve the accuracy of SM-miRNA association prediction, we designed two sets of experimental conditions for SMMA-HNRL. One set only contained heterogeneous information of three networks (SM-miRNA association network, integrated SM similarity network and integrated miRNA similarity network). There were only two kinds of nodes (miRNAs and SMs) in this heterogeneous network which was named as HIN-2N. The other set contained the heterogeneous information of all six networks which included three kinds of nodes (miRNAs, diseases and SMs) and was named as HIN-3N. The experimental results (AUC, AUPR) were shown in Figure 6. The AUC and AUPR values of HIN-3N are 0.9875 and 0.9885, which are both higher than those of HIN-2N. The comparison results for the remaining metrics are listed in Supplementary Material S4, and results showed they were all enhanced with different degree. This fully confirmed that introducing disease association information in our study is more reliable and more effective in predicting potential SM-miRNA associations.

### Robustness testing

**FIGURE 7:**
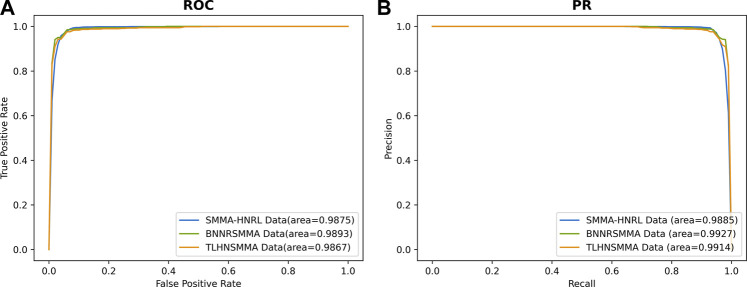
The ROC curves **(A)** and PR curves **(B)** of SMMA-HNRL in robustness testing.

The robustness is the ability of one predictive model to maintain a stable performance on different scales and types of datasets. To evaluate the stability of SMMA-HNRL, two datasets were downloaded from BNNRSMMA (Chen et al., 2021) and TLHNSMMA (Qu et al., 2018) respectively as independent validation datasets. The BNNRSMMA dataset included 831 SMs, 541 miRNAs and contained 664 pairs of SM-miRNA associations. The TLHNSMMA dataset included 831 SMs, 541 miRNAs and 383 diseases. There were 664 SM-miRNA association pairs and 6233 miRNA-disease association pairs in it. The two datasets also include integrated similarities of SMs, miRNAs and diseases. In 10-fold cross validation of the two independent validation sets, all parameters of SMMA-HNRL were the same in both testing. AUC and AUPR values of SMMA-HNRL are shown in Figure 7, and the comparison of other metrics is shown in Supplementary Material S5. The results indicated that SMMA-HNRL achieved promising results on two sets and had power robust to different SM-miRNA datasets.

### Independent test set validation

NoncoRNA database (Li et al., 2020) systematically recorded the information of ncRNAs with drug targets. Experimentally validated SM-miRNA interactions from NoncoRNA database were screened in our study and performed the same data preprocessing like those from SM2miR database. The data duplicated with SM2miR were eliminated with manual inspection. Finally, a total of 584 associations were obtained, including 272 miRNAs and 49 SMs. This data was not involved in the training of the model but as an independent test set to evaluate the generalization ability of SMMA-HNRL.

The ROC and PR curves of the experimental results of the independent test set which was exhibited in Supplementary Material S6. In the independent test set validation, the AUC and AUPR reached 0.9859 and 0.9859, respectively. This indicated that our model had a strong generalization capability, and the outperformance of SMMA-HNRL was not caused by overfittings.

### Model contrast

**FIGURE 8:**
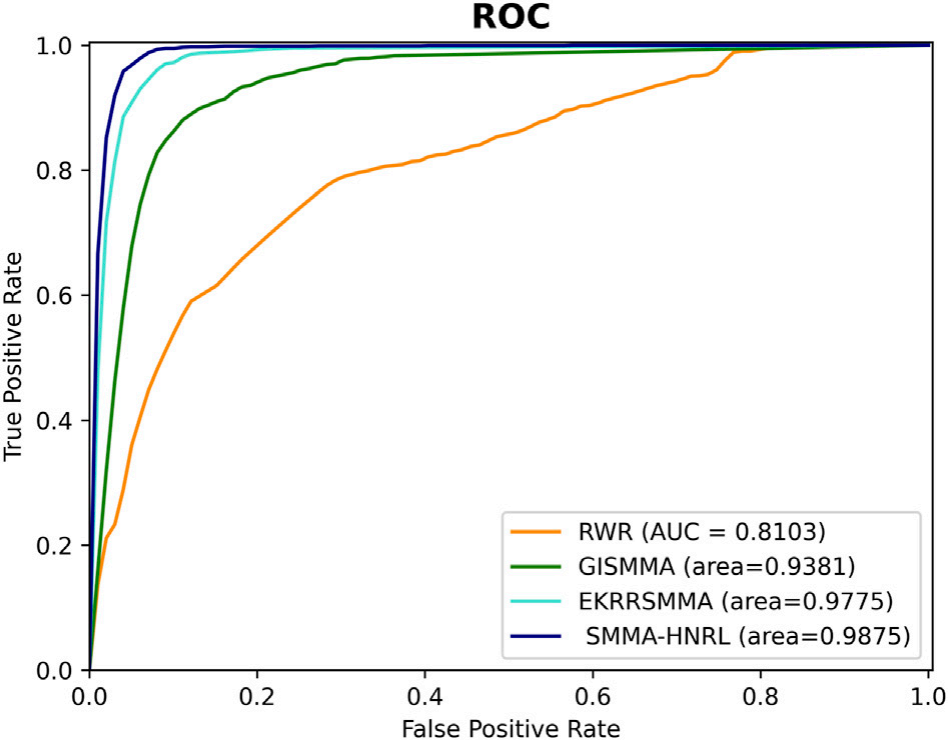
The ROC curves of SMMA-HNRL, RWR, GISMMA, and EKRRSMMA models.

To further demonstrate the predictive effectiveness of SMMA-HNRL, we compared SMMA-HNRL with the three state-of-the-art SM-miRNA association prediction models, EKRRSMMA (Wang et al., 2022), GISMMA (Guan et al., 2018) and RWR (Lv et al., 2015). In the contrast experiment, each model was trained and tested with the same datasets. The overview of datasets involved in each comparison model is exhibited in Supplementary Material S7. The prediction performance of the three models were performance with 10-fold cross validations, and the ROC curves were shown in Figure 8. It showed that SMMA-HNRL achieved AUC score of 0.9875, which outperformed RWR (AUC score: 0.8103), GISMMA (AUC score: 0.9381) and EKRRSMMA (AUC score: 0.9775). The merged feature vectors of SM nodes and miRNA nodes which were obtained with two different heterogeneous network representation learning algorithms (HeGAN and HIN2Vec) can improve prediction accuracy of potential SM-miRNA associations.

### Case studies

To further evaluate the capability of SMMA-HNRL in practical applications, we conducted case studies with three common SM drugs, 5-FU (DB00544), Cisplatin (DB00515) and Imatinib (DB00619), which were all closely related to human life and health.

First, the known SM-miRNA association vectors were used as positive samples and an equal amount of unknown associations that randomly generated was adopted as negative samples. Subsequently, SMMA-HNRL was trained with those samples. Then, miRNAs unrelated to the three SMs were screened from the heterogeneous network, and the SM-miRNA feature vectors were generated by Hadamard function. Finally, the feature vectors were input into the LightGBM classifier, and the probability scores were calculated. The predicted SM-miRNA associations were sorted in descending order by the probability scores. The potential associations were verified by manually reviewing the PubMed database for proving the effectiveness of SMMA-HNRL. The specific results were shown in the following table (Tables 5, 6, 7), and the full results for these three SMs are available in Supplementary Materials S8, S9, and S10.

**TABLE 5:** Validation of the top 50 predicted miRNAs related to 5-FU (DB00544).

miRNA	Evidence	miRNA	Evidence
hsa-mir-135a	29,735,329	hsa-mir-139	27,173,050
hsa-mir-150	32,669,857	hsa-mir-133b	32,865,180
hsa-mir-181a	29,795,190	hsa-mir-134	34,168,463
hsa-mir-214	30,915,129	hsa-mir-130a	30,510,209
hsa-mir-29b	34,155,879	hsa-mir-130b	33,816,278
hsa-mir-30c	Unconfirmed	hsa-mir-30d	Unconfirmed
hsa-mir-320	25,446,103	hsa-mir-30b	Unconfirmed
hsa-mir-328	33,948,374	hsa-mir-29c	31,037,126
hsa-mir-425	32,158,234	hsa-mir-26a	29,719,405
hsa-mir-451	Unconfirmed	hsa-mir-28	30,762,286
hsa-mir-96	31,089,750	hsa-mir-24-1	Unconfirmed
hsa-mir-98	Unconfirmed	hsa-mir-212	32,862,489
hsa-mir-16-1	Unconfirmed	hsa-mir-22	25,449,431
hsa-mir-363	27,167,197	hsa-mir-221	27,726,102
hsa-mir-335	31,799,650	hsa-mir-223	Unconfirmed
hsa-mir-324	30,103,475	hsa-mir-20b	27,878,272
hsa-mir-326	26,239,225	hsa-mir-205	32,996,748
hsa-mir-424	33,793,771	hsa-mir-199b	32,580,513
hsa-mir-378	30,797,151	hsa-mir-100	Unconfirmed
hsa-mir-181a-2	Unconfirmed	hsa-let-7f-2	Unconfirmed
hsa-mir-181b	27,081,844	hsa-let-7	26,687,759
hsa-mir-186	Unconfirmed	hsa-let-7c	33,051,247
hsa-mir-193b	34,844,630	hsa-mir-1-1	Unconfirmed
hsa-mir-148b	Unconfirmed	hsa-mir-1-2	Unconfirmed
hsa-mir-144	32,162,886	hsa-mir-124-1	Unconfirmed

**TABLE 6:** Validation of the top 50 predicted miRNAs related to Cisplatin (DB00515).

miRNA	Evidence	miRNA	Evidence
hsa-mir-302b	26,623,722	hsa-mir-26a	26,458,859
hsa-mir-181a-2	34,815,714	hsa-mir-22	30,537,795
hsa-mir-186	32,284,740	hsa-mir-26b	31,686,855
hsa-mir-452	Unconfirmed	hsa-mir-30b	33,779,882
hsa-mir-9-3	Unconfirmed	hsa-mir-328	30,221,716
hsa-mir-191	32,803,782	hsa-mir-326	26,239,225
hsa-mir-29b-2	Unconfirmed	hsa-mir-320	Unconfirmed
hsa-mir-1-1	Unconfirmed	hsa-mir-144	31,017,720
hsa-mir-24	30,787,983	hsa-mir-145	31,821,542
hsa-mir-34b	33,720,323	hsa-mir-140	32,765,679
hsa-mir-193b	27,918,099	hsa-mir-134	Unconfirmed
hsa-mir-194	32,534,701	hsa-mir-132	31,906,769
hsa-mir-200a	32,256,108	hsa-mir-125a	33,777,215
hsa-mir-206	27,014,910	hsa-mir-127	Unconfirmed
hsa-mir-139	33,300,085	hsa-mir-210	30,957,179
hsa-mir-143	33,090,550	hsa-mir-212	Unconfirmed
hsa-mir-495	34,747,666	hsa-mir-193a	30,485,589
hsa-mir-7	33,072,745	hsa-mir-15a	26,314,859
hsa-mir-99b	30,984,249	hsa-mir-483	Unconfirmed
hsa-mir-10b	32,892,697	hsa-mir-486	32,527,702
hsa-mir-1	32,377,691	hsa-mir-99a	27,994,509
hsa-mir-122	27,874,954	hsa-let-7f-2	Unconfirmed
hsa-let-7a	29,565,706	hsa-let-7g	Unconfirmed
hsa-mir-92-1	Unconfirmed	hsa-let-7d	30,816,441
hsa-mir-25	27,743,413	hsa-let-7f	26,458,859

**TABLE 7:** Validation of the top 50 predicted miRNAs related to Imatinib (DB00619).

miRNA	Evidence	miRNA	Evidence
hsa-mir-34a	31,923,418	hsa-mir-26b	31,273,251
hsa-mir-155	30,459,357	hsa-let-7b	Unconfirmed
hsa-mir-21	28,190,319	hsa-let-7c	Unconfirmed
hsa-mir-221	30,516,071	hsa-mir-191	Unconfirmed
hsa-mir-145	Unconfirmed	hsa-mir-93	Unconfirmed
hsa-mir-125b-2	Unconfirmed	hsa-mir-99b	28,544,907
hsa-mir-204	Unconfirmed	hsa-mir-197	Unconfirmed
hsa-mir-107	Unconfirmed	hsa-mir-127	Unconfirmed
hsa-mir-92a-1	Unconfirmed	hsa-mir-27a	26,458,312
hsa-let-7i	28,512,058	hsa-mir-151a	28,544,907
hsa-mir-223	32,597,702	hsa-let-7e	33,066,614
hsa-mir-224	26,458,312	hsa-mir-424	25,697,481
hsa-mir-24	Unconfirmed	hsa-mir-30b	Unconfirmed
hsa-mir-18a	26,458,312	hsa-mir-222	30,396,237
hsa-mir-125b	Unconfirmed	hsa-mir-205	28,861,326
hsa-let-7a	Unconfirmed	hsa-mir-206	Unconfirmed
hsa-mir-148a	Unconfirmed	hsa-mir-373	Unconfirmed
hsa-mir-25	Unconfirmed	hsa-let-7	Unconfirmed
hsa-mir-27b	28,942,039	hsa-mir-494	28,533,480
hsa-mir-200a	28,942,039	hsa-mir-483	34,638,938
hsa-mir-19a	28,942,039	hsa-mir-34c	Unconfirmed
hsa-mir-1	Unconfirmed	hsa-mir-125b-1	Unconfirmed
hsa-mir-152	Unconfirmed	hsa-mir-200b	Unconfirmed
hsa-mir-15b	Unconfirmed	hsa-mir-22	Unconfirmed
hsa-mir-29b-1	31,923,418	hsa-mir-214	28,942,039

5-FU, a pyrimidine analog, is a key chemotherapeutic drug in colorectal cancer (CRC) and has been implicated in the treatment of breast cancer. As an antimetabolite, it interferes with DNA synthesis by blocking the conversion of deoxyuridine to thymidylate by thymidylate synthase (Longley et al., 2003; Wigmore et al., 2010). Table 5 showed the top 50 miRNA associations associated with 5-FU, among the top 10, 8 miRNAs were confirmed by the literature, among the top 30, 23 miRNAs were confirmed by the literature, and among the top 50, 34 miRNAs were confirmed by the literature.

Cisplatin, the first metal-based anticancer drug, is widely used to treat various types of cancers, such as testicular cancer, ovarian cancer, lung cancer. It induces DNA damage by interacting with purine bases on DNA and eventually induces cancer cell apoptosis (Ghosh, 2019). Table 6 presented the top 50 miRNA associations associated with cisplatin, 6 miRNAs of the top 10, 25 miRNAs of the top 30 and 37 miRNAs of the top 50 were documented confirmed by the literature.

Imatinib is a potent drug for chronic myeloid leukemia, which inhibits the rapid division of cancer cells by inhibiting specific tyrosine kinases (Peng et al., 2005). Table 7 showed the top 50 miRNA associations associated with imatinib, 5 miRNAs of the top 10, 15 miRNAs of the top 30 and 22 miRNAs of the top 50 were confirmed by the literature.

In summary, through the case studies of 5-FU, Cisplatin and Imatinib, the majority of novel associations with the highest probability has been confirmed by the PubMed literatures, and it is enough to illustrate the outstanding performance of SMMA-HNRL in predicting potential SM-miRNA associations.

## Discussion

Numerous studies proved that many human complex diseases are closely related to the dysregulations of related key miRNAs, and miRNAs have been recognized as a potential class of drug targets. Predicting novel SM-miRNA associations is important to help researchers find effective drugs, understand the molecular basis of diseases and reduce experimental costs. Nowadays, it has become a trend to construct heterogeneous networks to predict potential SM-miRNA associations by integrating multiple biological entities. In this work, we proposed a novel model SMMA-HNRL based on an integrated heterogeneous network representation learning algorithm. By building a heterogeneous information network with the HeGAN algorithm based on the generative adversarial network and the HIN2Vec algorithm based on the random walk of the meta-path, richer feature information of the heterogeneous network was accessed, which overcame the data sparsity due to few known associations. Validated by the experiments, SMMA-HNRL exhibited high robustness. Compared with three state-of-the-art predicting models, our model achieved the best performance under the same dataset evaluated by 10-fold cross validation. Case studies of three common drugs showed that the model had good application significance. Otherwise, SMMA-HNRL can be regarded as an open framework, and users can adopt more heterogeneous information related with SM-miRNA association prediction for improving prediction accuracy.

Although SMMA-HNRL has achieved satisfactory results in predicting potential SM-miRNA associations, there is still room for improvement in the experiments. First, due to the current biological limitations, we do not exactly find negative samples of SM-miRNA associations, so future research will introduce more effective negative sample screening methods. Second, although this study introduced multi-source heterogeneous data for network construction, it still cannot fully reflect the comprehensive and complex interaction network. The more data integrated, the higher the accuracy and robustness of the model will be. In the future, more biological data will be introduced for processing, such as lncRNA and gene related data.

## Data Availability

The original contributions presented in the study are included in the article/Supplementary Material, further inquiries can be directed to the corresponding author.
