# Supplementary material for: Prediction of potential small molecule−miRNA associations based on heterogeneous network representation learning
Source: Front Genet. 2022 Dec 2;13:1079053. doi: 10.3389/fgene.2022.1079053 (PMC9755196; doi:10.3389/fgene.2022.1079053)
Supplement: Supplementary file 2 [file Table1.DOCX]

**The Summarization of SM-miRNA Prediction Models**

| Categories | PMID | Model name | Introduction |
| --- | --- | --- | --- |
| Models based on biological networks | 26198104 | RWR | SM-miRNA association prediction based on Random Walk with Restart (RWR) algorithms. |
|  | 27329603 | SMiR-NBI | A network-based inference framework for SM-miRNA association prediction. |
|  | 29943160 | TLHNSMMA | A SM-miRNA association prediction framework based on a triple layer heterogeneous network and finally predicted potential associations by the iterative update algorithm based on the global network. |
|  | 30374302 | GISMMA | A novel computational model based on Graphlet interactions and a linear regression was performed between the number of different types of Graphlet interactions and the SM-miRNA association scores to calculate the final scores. |
|  | 33166451 | SMMART | Graph regularization technique for the prediction of SM-miRNA associations. |
| Models based on machine learning algorithms | 30840454 | RFSMMA | A filtering approach was employed to extract reliable features of SM-miRNA pairs by using their similarity data. Subsequently, random forest algorithm was used for SM-miRNA association prediction. |
|  | 34676393 | EKRRSMMA | By constructing different feature subsets for SMs and miRNAs, an integrated learning model containing multiple KRR-based base learning tasks was constructed. The prediction results of all base learners were averaged and the final results were introduced as the SM-miRNA association scores. |
| Other prediction models | 28692985 | EmDL | A new text mining framework for extracting associations between miRNAs and SMs efficacy from the literature and recording them in the database |
|  | 22355792 | SMirN | A SM-miRNA Network for each type of 23 common cancers was constructed by collecting relevant data. The associations of cancer-related miRNAs with SMs were determined by the enrichment scores |
