# Supplementary figures and images for: Prediction of potential small molecule−miRNA associations based on heterogeneous network representation learning
Source: Front Genet. 2022 Dec 2;13:1079053. doi: 10.3389/fgene.2022.1079053 (PMC9755196; doi:10.3389/fgene.2022.1079053)

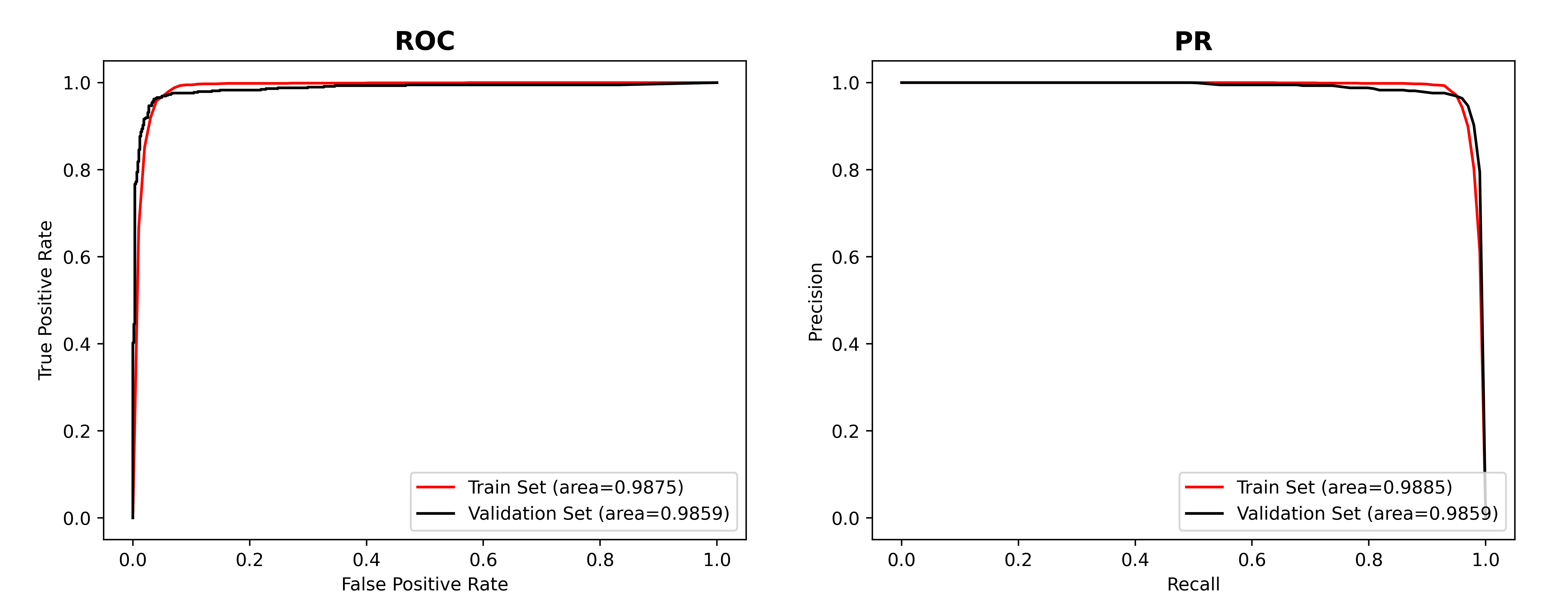

Supplement: Supplementary file 4 [file Image1.TIF]
