# Supplementary material for: Prediction of potential small molecule−miRNA associations based on heterogeneous network representation learning
Source: Front Genet. 2022 Dec 2;13:1079053. doi: 10.3389/fgene.2022.1079053 (PMC9755196; doi:10.3389/fgene.2022.1079053)
Supplement: Supplementary file 6 [file Table2.DOCX]

**Experimental code details of SMMA-HNRL**

**Part 1. Details of cross-validation experiments:**

Number of positive samples: 1766

Number of negative samples: 1766

10-fold cross validation:

Train Set Samples: Validation Set Samples = 3178:354

All classifiers' parameters were set as defaults in scikit-learn packages.

**Part 2. Details of independent validation experiments:**

Number of positive samples: 584

Number of negative samples: 584

All classifiers' parameters were set as defaults in scikit-learn packages.

**Part 3. Python and packages versions for SMMA-HNRL**

| **Python and Package Name** | **Python and Package Version** |
| --- | --- |
| Python | 3.8.x |
| networkx | 2.0 |
| numpy | 1.20.1 |
| pandas | 1.2.4 |
| scikit-learn | 0.24.1 |
| scipy | 1.6.2 |
| tensorflow | 1.14.0 |
